# What have we learned in interventional pulmonology in the past decade?

**DOI:** 10.3906/sag-1904-20

**Published:** 2019-10-24

**Authors:** Mehmet Akif ÖZGÜL, Erdoğan ÇETİNKAYA, Demet TURAN, Efsun Gonca UĞUR CHOUSEIN, Deniz DOĞAN, Ekrem Cengiz SEYHAN

**Affiliations:** 1 Department of Pulmonology, Yedikule Chest Diseases and Thoracic Surgery Training and Research Hospital, Health Sciences University, İstanbul Turkey; 2 Department of Pulmonology, Gulhane Training and Research Hospital, Health Sciences University, Ankara Turkey

**Keywords:** Experience, progress, interventional pulmonology

## Abstract

**Background/aim:**

The increasing number of lung diseases and particularly pulmonary malignancies has intensified the need for diverse interventions in the field of interventional pulmonology. In recent years we have seen many new developments and expanding applications in the field of interventional pulmonology. This has resulted in an increased number and variety of performed procedures and differing approaches. The purpose of the present study is to provide information on patient characteristics, range of interventions, complication rates, and the evolving approach of an experienced center for interventional pulmonology.

**Materials and methods:**

We retrospectively examined the records of 1307 patients who underwent a total of 2029 interventional procedures in our interventional pulmonology department between January 2008 and December 2017.

**Results:**

About half of the interventional procedures (47.2%) were performed on patients with airway stenosis due to malignant disease. Among patients with benign airway stenosis, the most frequent reason for intervention was postintubation tracheal stenosis. The number of patients who developed complications was 81 (6.2%), and the most common complication was hemorrhage (n = 31, 2.99%); 94.9% (n = 1240) of interventional procedures were performed under general anesthesia, without complications or deaths associated with anesthesia. Only one death (0.076%) occurred in the perioperative period. A total of 18 patients (1.38%) died in the 30-day perioperative and postoperative period. None of the patients with benign airway stenosis died.

**Conclusion:**

Interventional bronchoscopy is an invasive but considerably safe and efficient procedure for selected cases and effective treatment modality for airway obstructions, massive hemoptysis, and foreign body aspiration. Interventional pulmonology is a field of pulmonary medicine that needs effort to progress and provide an opportunity to witness relevant developments, and increase the number of competent physicians and centers.

## 1. Introduction

The first bronchoscopes used in medicine were rigid bronchoscopes. Interventional bronchoscopy was first performed by Gustav Killian in 1897. Dr. Killian used bronchoscopy to successfully remove a bone from the right main bronchus of a German farmer [1]. The rod-lens telescope of the contemporary rigid bronchoscope was developed by Harold Hopkins and modified to reach its final design by Karl Storz [2]. Advances in the field of bronchoscopy accelerated following the invention of the flexible bronchoscopy by Shigeto Ikeda in the second half of the 20th century [3]. Today, the main areas of application of interventional bronchology are malignant airway obstructions (MAO) primarily secondary to lung cancer, and benign airway obstructions (BAO) resulting from postintubation and posttracheostomy tracheal stenosis [4,5]. 

Additionally, bronchoscopic lung volume reduction (BLVR) and transbronchial cryobiopsy (TBCB) are additional applications of bronchoscopy which are evolving [6,7]. The purpose of this study is to share our 10-year experience in interventional pulmonology. In this regard, we tried to determine the range of procedures performed, the demographic characteristics of patients, and complication rates. 

## 2. Materials and methods

### 2.1. Study design

The objective of this study was to retrospectively analyze the data for the advanced interventional pulmonary procedures we have performed over the last 10 years. The primary objective was to examine the range of cases and interventions, and to assess our complication and mortality rates.

### 2.2. Study population

Following approval of the local ethics committee, we retrospectively examined the records of patients between January 2008 and December 2017. Patients who underwent interventional procedures in our interventional pulmonology department were included in the study. Our hospital is a tertiary referral center with the highest patient volume in Turkey. Medical records of all patients who underwent advanced bronchoscopic intervention, medical thoracoscopy, or whole-lung lavage therapy for pulmonary alveolar proteinosis were recorded and reviewed. Bronchoscopic interventions were assigned into two groups according to whether rigid or fiberoptic bronchoscopic interventions were used. The rigid bronchoscopic intervention group was further divided into subgroups according to the primary indications (malignant and benign airway obstructions, foreign body aspiration, hemoptysis, etc.). For fiberoptic bronchoscopic procedures, only patients who underwent cryotransbronchial biopsy or who were treated with bronchoscopic lung volume reduction were included in the study. For each group, we recorded demographic data such as patients’ age and sex, interventions performed, number of procedures, and complication rates. Mortality rates were calculated based on data of patients who died perioperatively and within 30 days of the procedure. Patient consent forms were obtained from all patients due to the general practice of our hospital. Approval was obtained from Yedikule Chest Diseases and Thoracic Surgery Training and Research Hospital Scientific Committee with the code: 2019: 226/3

### 2.3. Interventional procedures

Fiberoptic bronchoscopic procedures (BF-1TQ180 and BF-XP 160, Olympus, Tokyo, Japan) were performed under conscious sedation with a short-acting benzodiazepine (midazolam). Rigid bronchoscopic procedures (Karl Storz, Germany) were performed under general anesthesia (total intravenous anesthesia), while medical thoracoscopic procedures were performed with the same bronchoscope under conscious sedation. In addition, fluoroscopy was used for transbronchial cryobiopsy (TBCB) and coil procedures. Midazolam (2–5 mg) was the anesthetic used for bronchoscopic procedures done under conscious sedation (moderate sedation). Following local anesthesia of the oronasal airway with 10% lidocaine spray/gel, the bronchoscope was inserted. When the nasal route was not accessible, the oral route was used. Lidocaine 1% was applied as needed throughout the procedure after passing the glottis using the SAYGO (spray-as-you-go) technique. All patients were routinely monitored with electrocardiography and SpO2 during the procedure, with arterial blood pressure measured every 5 min. General anesthesia induction was achieved with midazolam 0.05–0.10 mg/kg, propofol (maximum dose 1000 mg), remifentanil (maximum dose 2 mg), and rocuronium (maximum dose 50 mg), dosage given according to the patient’s condition. We did not encounter any side effects or complications due to anesthesia. 

## 3. Results

### 3.1. General findings

Over the period of 10 years, the mean age of the study population was 55.9 ± 14.4 with 74% (968) being male. About half (47.2%) of the interventional procedures were performed to treat malignant airway obstructions (MAO). The lowest procedure rate of 1% was for patients with stenosis following lung transplantation. Table 1 summarizes the distribution ratios of the patients according to performed interventions and indications.

**Table 1:** Distribution of interventional procedures according to indications.

Indications			n (%)
Procedures withrigid bronchoscopy	Malignant stenosis	Malignant airway obstructions (MAO) secondary to lung carcinomas	550 (42.08)
		MAO secondary to endobronchial metastasis/invasion	67 (5.12)
	Benign stenosis	Airway obstruction secondary to benign tumors	46 (3.52)
		Postintubation tracheal stenosis (PITS)	111 (8.5)
		Fibrostenosis	20 (1.53)
		Posttransplantation stenosis	13 (1)
		Tracheobronchomalacia (TBM)	18 (1.38)
		Posttracheostomy tracheal stenosis (PTTS)	26 (1.99)
	Diagnostic bronchoscopy		144 (11.02)
	Hemoptysis		106 (8.11)
	Foreign body aspiration		35 (2.67)
Fiber optic bronchoscopy (FOB)	Transbronchial cryobiopsy (TBCB)		50 (3.82)
	Bronchoscopic lung volume reduction (BLVR) (EBV and coil)		67 (5.13)
Other	Medical thoracoscopy (MT)		37 (2.83)
	Pulmonary alveolar proteinosis (PAP)		17 (1.3)
Total			1307 (100)

As for the demographic data, patients with tracheobronchomalacia (TBM) had the highest average age of 67.7 ± 11.5, while patients who were treated with whole-lung lavage for pulmonary alveolar proteinosis had the lowest average age of 34.8 ± 8.7. Patients with posttracheostomy tracheal stenosis (PTTS) had the highest rate of procedures per case (2.88 ± 3.01). While 94.9% (n = 1240) of interventional procedures were performed under general anesthesia, only endobronchial valves and medical thoracoscopy (MT) procedures were performed under conscious sedation with midazolam. Endobronchial stents were placed only for airway obstructions of either malignant or benign etiology. There were 851 patients in these two groups, of whom 283 (33.25%) received stent placement; the majority of stents (73.1% vs 26.9%) were placed in patients with malignant airway obstruction (Table2).

**Table 2:** Demographic features of the study population.

Categories		n	Age (Year)	Female/ Male	Number of procedures	Procedure number per case	Type of anesthesia	Numberof stents %
Malignant stenosis	Lung tumors	550	58.0 ± 11.8	82/468	834	1.51 ± 1.06	GA	176/32
	Metastasis/ invasion	67	58.7 ± 14.2	34/33	119	1.77 ± 1.27	GA	31/46.3
Benign stenosis	Benign tumors	46	53.2 ± 15	11/35	82	1.78 ± 1.28	GA	…
	Postintubation tracheal stenosis (PITS)	111	53.4 ± 17	54/57	281	2.51 ± 2.56	GA	50/46.8
	Fibrostenosis	20	44.2 ± 10.5	6/14	54	2.70 ± 3.37	GA	6/30
	Posttransplantation stenosis	13	42.7 ± 16.3	2/11	25	1.92 ± 1.11	GA	3/2.3
	Tracheobronchomalacia	18	67.7 ± 11.5	7/11	46	2.55 ± 1.75	GA	1583.4
	Posttracheostomy tracheal stenosis	26	55 ± 14.5	12/14	75	2.88 ± 3.01	GA	20.8
Interventional diagnostic bronchoscopies		144	58.4 ± 13.9	43/101	164	1.32 ± 0.47	GA	…
Hemoptysis		106	52.8 ± 16.9	20/86	106	1	GA	…
Foreign body aspiration		35	50.3 ± 19.7	13/22	35	1	GA	…
Transbronchial cryobiopsy		59	53.5 ± 13.7	30/20	59	1	GA	…
Bronchoscopic lung volume reduction		67	62.8 ± 7.3	7/60	67	1	GA*/MS	…
Medical thoracoscopy		37	50.94 ± 15.38	11/26	37	1	MS	…
Pulmonary alveolar proteinosis		17	34.8 ± 8.7	7/10	45	2.64 ± 1.45	GA	...

Abbreviations: PITS = Postintubation tracheal stenosis, PTTS = Posttracheostomy Tracheal stenosis, TBM = Tracheobronchomalacia, FBA = Foreign body aspiration, PAP = Pulmonary alveolar proteinosis. BLVR = Bronchoscopic lung volume reduction, TBCB = Transbronchial cryobiopsy, MT = Medical thoracoscopy, GA = General anesthesia, MS = Moderate sedation, *Coil procedure.

The complication rate was 6.2% (n = 81) for the entire study population, with the most common complication being hemorrhage with a rate of 2.99% (n = 31). A total of 18 patients died and the 30-day peri-/postoperative mortality rate was found to be 1.38%. While interventions performed by rigid bronchoscopy for malignant or benign disorders had a complication rate of 4.59%, most deaths occurred in the patients who underwent interventional procedures for MAO. One of the patients with nonsmall cell lung cancer died perioperatively from cardiac arrest and 15 patients died from severe respiratory failure while being treated in the intensive care unit. Only 2 deaths resulted from the remaining procedures. One patient died from massive hemoptysis on the eighth day after coil placement, and the second patient died from respiratory failure resulting from primary disease progression (Table 3).

**Table 3:** Procedures with complications and 30-day mortality rates.

Complications	Malignant stenosis	Benign stenosis	Bronchoscopic lungvolume reduction (BLVR)	Medicalthoracoscopy (MT)	TransbronchialCryobiopsy (TBCB)
Hemorrhage, %	14/2.26	2/8.5	…	…	15/30
Arrhythmias, %	12/1.95	5/2.1	…	…	…
Prolonged intubation duration, %	3/0.48	…	…	…	…
Pneumothorax (Px), %	…	…	…	…	6/12
Exitus, %	16*/2.59	…	1/1.5	…	1/2
Others	…	…	…	6	…
Total, %	52/4.59		29/17.0		

### 3.2. Malignant airway obstructions

**Figure 1:**
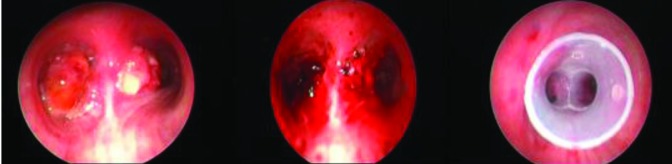
Malignant airway obstruction (MAO) in left and right main bronchus, before and after endobronchial therapy and silicone Y-stent application.

**Figure 2:**
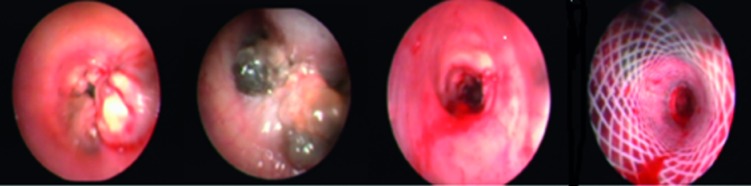
MAO in the trachea secondary to malignant melanoma, before and after endobronchial therapy poliflex stent insertion.

A total of 617 patients underwent 953 interventional procedures for MAO. This group of patients had a complication rate of 7.3% and a mortality rate of 2.59%. Pulmonary malignancies were the most common cause of MAO (Figures 1 and 2). The histopathological results of 27 patients who were referred to our center from different centers were not available for review. The most common diagnosis (42.3%) of patients whose pathology reports were available was squamous cell lung carcinoma (Table 4). Regarding the serious complications of the procedure, 1 patient died of perioperative cardiac arrest, 8 patients required admission to the intensive care unit because of prolonged intubation after the procedure, and 15 of these patients died within a month. There were 119 interventional procedures performed on 67 patients with airway obstruction secondary to endobronchial metastasis or invasion of extrapulmonary malignancies. The most frequent causes of MAO in these patients were esophageal, thyroid, and renal cell carcinomas. Procedure-related complications were seen in 7 patients, the most serious being prolonged bleeding in a patient with renal cell carcinoma. None of these patients died perioperatively or in the early postoperative period.

**Table 4:** Histopathologic types and percentages of lung tumors.

Histopathogy	n (%)
Squamous cell carcinoma	221 (42.3%)
Nonsmall cell lung carcinoma	151 (28.9%)
Small cell lung carcinoma	47 (9.0%)
Adenocarcinoma	39 (7.5%)
Carcinoid tumor	45 (8.6%)
Adenoid cystic carcinoma	6 (1.1%)
Carcinoma in-situ	4 (0.8%)
Low differentiated carcinoma	3 (0.6%)
Malignant mesenchimal tumor	3 (0.6%)
Low grade neuroendocrine tumor	2 (0.4%)
High grade neuroendocrine tumor	1 (0.2%)
Papillary malignant epithelial tumor	1 (0.2%)
Total	523 (100.0%)

* Pathology reports of 27 patients could not be found.

### 3.3. Benign airway obstructions

**Figure 3:**
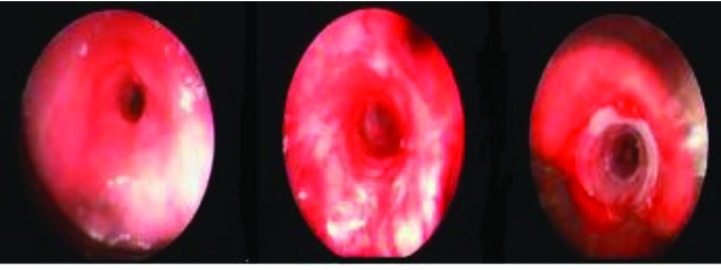
Benign airway obstruction (BAO), complex type tracheal stenosis, after mechanical dilation and silicone tracheal stenotic stent application.

**Figure 4:**
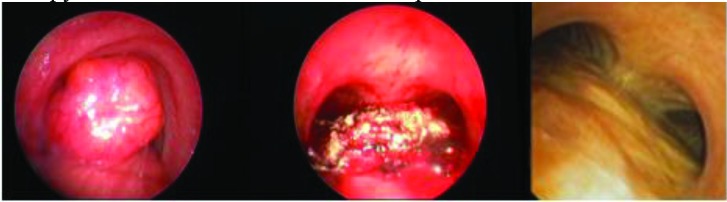
BAO secondary to pulmonary amiloidoma on the main carina after endobronchial therapy, and view after 5 months follow-up.

Two hundred thirty-four patients with benign airway obstruction (BAO) underwent rigid bronchoscopy under general anesthesia. Of them 46 had benign tumors, 111 had postintubation tracheal stenosis, 20 had fibrostenosis, 13 had posttransplantation stenosis, 18 had tracheobronchomalacia, and 26 had posttracheostomy tracheal stenosis (Table 2). Among all BAO, debulking and subsequent histopathological evaluation revealed 46 benign tumors. Of these, the most common diagnosis was hamartoma (30.4%). During the period of 10 years, there were 281 procedures performed on 111 patients with postintubation tracheal stenosis (PITS), the number of procedures per patient averaging 2.51 ± 2.56. The average duration of symptoms before treatment was 7 ± 6.9 weeks and the average duration of intubation was 15.2 ± 9.3 days. Eighty-four patients (75.7%) had complex-type stenotic lesions (Figures 3 and 4). Fifty patients (46.8%) were treated with stent implantation, the most preferred (96%) type being the stenotic tracheal stent. No complications were observed related to the interventions. The highest numbers of procedures per patient were performed on the group of patients with PTTS. In this group, 75 interventional procedures were performed on 26 patients (average number of procedures per case 2.88 ± 3.01) without any complications. Other causes of benign stenosis were fibrostenosis at the anastomosis line following surgery in 20 patients, stenosis following lung transplantation in 13 patients, and 18 patients who had been operated on for tracheobronchomalacia. None of the patients who underwent interventional procedures for benign airway obstruction died.

### 3.4. Medical thoracoscopy (MT)

A total of 37 patients (26 M, 11 F) underwent diagnostic MT. These patients had a failed diagnosis by classical methods, including thoracentesis and closed pleural biopsy, before medical thoracoscopy. Among all patients, the diagnoses were pleural effusion due to tuberculosis (n = 13), malignant pleural mesothelioma (n = 5), nonsmall cell lung cancer metastasis (n = 2), small cell lung cancer metastasis (n = 1), breast cancer metastasis (n = 1), renal cell cancer metastasis (n = 2), larynx cancer metastasis (n = 1), primary pleural B-cell lymphoma (n = 1), benign pleural asbestosis (n = 1), and finally, chronic nonspecific pleuritis (n = 9). In one patient, a diagnosis was not achieved after MT. The complications rate was 19% (n = 7). Four patients had subcutaneous emphysema and 3 patients required chest tube drainage for lung nonexpansion. One patient with prolonged air leakage died on day 34 postintervention secondary to severe respiratory failure.

### 3.5. Transbronchial cryobiopsy (TBCB)

Transbronchial cryobiopsy (TBCB) was performed on 59 patients with an adequate pathologic specimen being obtained in 96%. A cryoprobe with a 1.9-mm external diameter was used. Biopsy was performed during general anesthesia and under fluoroscopic guidance. Biopsy material obtained had a mean tissue surface area of 31.8 ± 16 mm². One patient died (2%) following TBCB secondary to progression of his/her primary disease. The most common complications were pneumothorax requiring chest tube placement in 6 (12%) patients and hemorrhage in 15 patients (30%). Hemorrhage was graded according to the amount of bleeding and endoscopic procedures required to control bleeding [8]. Among patients who developed hemorrhage, 8 (53.3%) patients had grade I and 7 (46.7%) patients had grade II hemorrhage. 

### 3.6. Bronchoscopic lung volume reduction (BLVR): endobronchial valve (EBV) and coil treatments

A total of 67 patients underwent BLVR interventions (30 patients had EBV and 37 had coil treatment). EBV procedures were performed under conscious anesthesia with midazolam, and coil procedures were performed under general anesthesia with fluoroscopic guidance. The most important intervention-associated complication was the development of massive hemoptysis in 1 patient on the eighth day following coil treatment. This patient died of asphyxia secondary to hemoptysis.

### 3.7. Other procedures

One hundred fourteen patients underwent diagnostic rigid bronchoscopy either because fiberoptic bronchoscopy was nondiagnostic or because a high bleeding risk precluded biopsy. Thirty-five patients underwent foreign body aspiration and 17 patients were treated with whole-lung lavage for pulmonary alveolar proteinosis. A total of 106 patients underwent rigid bronchoscopy for hemoptysis evaluation. Of them, the majority of patients had endobronchial malignancies. Among these patients, none developed complications or died perioperatively or in the early postoperative period.

## 4. Discussion

In this retrospective study we presented our 10-year experiences in interventional bronchoscopy. During this period, 2029 interventional procedures were performed on 1307 patients; 81 patients suffered complications, corresponding to a rate of 6.2%. Eighteen patients died, with 30-day mortality being 1.38%. 

Lung cancer is the most common cause of MAO [9]. The majority of patients operated for malignant airway obstruction in our case series had lung cancer (seen in Table 1). A total of 953 interventional procedures were performed on 617 patients, with a complication rate of 7.3% and 30-day mortality of 2.59%. In their multicenter prospective study, Ernst et al. reported that the complication rate and 30-day mortality of 554 therapeutic procedures were 19.8% and 7.8%, respectively [10]. Among 253 patients with MAO, one died during the procedure, while 42% of cases were operated on on an emergent basis. In a study on therapeutic bronchoscopy for MAO with the participation of 15 centers, 1115 interventional procedures were performed on 947 patients; the complication rate was reported as 3.9%. However, the rate of complications differed to a great extent (from 0.9% to 11.7%) between centers. Consistent with our experience, 30-day mortality averaged 14.8%, with a range of 7.7% to 20.2% among the different centers [10,11]. The other group consisted of the patients with airway obstruction secondary to benign tumors. The majority of these patients had hamartoma, which is the most common benign tumor of the lungs [12]. The most frequent causes of benign airway obstruction were PITS and PTTS [13,14]. Likewise, in our series, patients with airway stenosis due to PTTS and PITS received the highest number of interventions and had the highest rate of interventions per patient. This was the result of the need for recurrent dilations and frequent follow-ups [10]. Our study included 301 patients who were treated for benign airway obstruction; of these, 22.2% had PITS and 83.6% were found to have complex types of tracheal stenosis. 

In our series, 281 interventional procedures were performed on 111 patients with PITS. Similarly, 84% of our patients had complex types of tracheal stenosis. Another patient group with a benign disorder causing airway obstruction was lung transplantation patients with stenosis developing at the anastomosis line; 25 interventional procedures were performed on 13 such patients without any complications. Studies have reported that stenosis at the anastomosis line is the most frequent complication developing in lung transplant patients, with rates of 13%–16% [15,16].

Life-threatening hemoptysis has been reported in 5%–15% of cases [17]. Mortality from massive hemoptysis ranges from 9% to 38%, the highest rate being reported in patients with advanced lung cancer [18]. A bleeding focus was identified and controlled successfully in 61.3% of patients who underwent rigid bronchoscopy for hemoptysis. In these patients, the most commonly observed etiology was endobronchial malignancy. Bronchoscopic examination accurately identified the bleeding focus in 73%–93% of patients with hemoptysis [19]. In experienced centers, rigid bronchoscopy is the intervention of choice because it confers advantages such as securing the airway and providing for ventilation, especially in the presence of massive hemoptysis, it has a stronger aspiration capacity thanks to its wide working channel, and allows the simultaneous use of a fiberoptic bronchoscope in order to reach the distal airways [20].

Medical thoracoscopy is a safe procedure with low mortality rate (0.35%) in experienced hands [21]. We encountered 4 cases of subcutaneous emphysema and 3 cases of prolonged air leakage. None of our patients had lung laceration, but one of the patients with prolonged air leakage died of respiratory failure 34 days after the procedure. In a study on the safety and complications of MT, the rate of nonserious complications was 16.5%, the most frequent being prolonged air leakage and fever, and there was no operative mortality. The most serious complication, pulmonary laceration, was encountered in 3 patients, leading the authors to conclude that better case management is needed for these patients [22].

The classical treatment of pulmonary alveolar proteinosis (PAP) is whole-lung lavage performed under general anesthesia and selective intubation [23]. As it carries a low risk of life-threatening complications, this method is considered a safe and effective treatment option in the management of PAP. The most frequent complication related to the procedure was reported to be transient elevation of temperature (18%), and the risk of pneumothorax was considerably low (0.8%) [24]. On the other hand, in a study with 12 participating centers, 11 of 33 patients (33%) undergoing whole-lung lavage experienced complications, the most common being oxygen desaturation. The same study reported that 2 patients died; one PAP patient secondary to myelodysplastic syndrome, and the other due to disease-associated adults’ respiratory distress syndrome (ARDS) [25]. We did not observe any perioperative or early postoperative complications in a total of 45 operations performed on 17 patients.

Pneumothorax and hemorrhage are the most frequently observed complications of TBCB [26]. A metaanalysis of data from 994 participants in 15 studies reported an average pneumothorax rate of 10% [27]. In another metaanalysis of 13 studies, the same rate was found to be 9.5% (5.9%–14.9%). Generally, the rates of pneumothorax, bleeding, and 30-day mortality following TBCB are reported to be 0%–26%, 0%–42%, and <2%, respectively; in our patient series, the respective rates were 12%, 30%, and 2% [28].

In recent years, we have witnessed many new developments and expanding applications in the field of interventional pulmonology. In the last 10 years, the number of procedures performed by our team has increased along with our experience, and our approach has evolved as new procedures have become available.

When considering our data, we saw that our approach to patients with tracheomalacia during this time period has evolved from stent placement towards CPAP titration, which is less invasive.

We also noticed that in the last 5 years of the study period, we have predominantly used silicon stents instead of metallic ones because of fewer complications and increased ease of removal.

In the last years, increased cost awareness has resulted in the use of domestically produced stents. Although their placement is more difficult and time consuming than that of original stents, our increasing experience has mitigated this problem.

While LASER was used more frequently in the first years of our study, APC, being cost effective and efficient, has been used increasingly more often during the latter years and has become as efficient and safe as LASER in regard to bleeding control and coagulation.

BLVR and TBCB are becoming routinely performed procedures, and with increasing experience we obtain better results.

Our team of physicians follow up with the patients according to predetermined charts and are able to detect and treat complications early.

We support the training of physicians from other centers in order to increase the number of patients that can benefit from interventional pulmonology.

The most important limitations of this study are its retrospective nature and, despite a large number of patients, being a single-center study. Nonetheless, with the high patient volume we believe that it contributes to the current English literature and gives valuable information regarding interventional pulmonology. 

In conclusion, with the present study we have shared our 10 years of experience, evolving approaches, and the inclusion of new modalities in the field of interventional pulmonology. Although invasive, interventional bronchoscopy is a safe and efficient procedure for selected cases, especially in experienced hands. It is an excellent treatment modality both for airway obstructions developing secondary to malignancies, particularly lung cancer, and for life threatening conditions such as massive hemoptysis and foreign body aspiration. 

Interventional pulmonology is a field of pulmonary medicine that should not be overlooked. Efforts should be made to advance this field and to increase the number of competent physicians and centers. We consider that disclosing practical experiences in this area of pulmonary medicine will both spark interest in interventional pulmonology and will provide an opportunity to witness relevant developments.
